# MiR-4763-3p targeting *RASD2*as a Potential Biomarker and Therapeutic Target for Schizophrenia

**DOI:** 10.14336/AD.2022.0103

**Published:** 2022-07-11

**Authors:** Jiao Wang, Wenxin Qi, Hongwei Shi, Lin Huang, Fujiang Ning, Fushuai Wang, Kai Wang, Haotian Bai, Hao Wu, Junyi Zhuang, Huanle Hong, Haicong Zhou, Hu Feng, Yinping Zhou, Naijun Dong, Li Liu, Yanyan Kong, Jiang Xie, Robert Chunhua Zhao

**Affiliations:** ^1^School of Life Sciences, Shanghai University, Shanghai, China.; ^2^School of Computer Engineering and Science, Shanghai University, Shanghai, China.; ^3^PET Center, Huashan Hospital, Fudan University, Shanghai, China.; ^4^Institute of Basic Medical Sciences Chinese Academy of Medical Sciences, School of Basic Medicine Peking Union Medical College, Beijing, China.; ^5^Centre of Excellence in Tissue Engineering, Chinese Academy of Medical Sciences, Beijing, China.; ^6^Beijing Key Laboratory of New Drug Development and Clinical Trial of Stem Cell Therapy (BZ0381), Beijing, China.; ^7^Central Laboratory, Shanghai Chest Hospital, Shanghai Jiao Tong University, Shanghai, China.; ^8^Psychological Rehabilitation Hospital of Penglai District, Yantai, Shandong, China

**Keywords:** Schizophrenia, *RASD2*, miR-4763-3p, Biomarker

## Abstract

Existing diagnostic methods are limited to observing appearance and demeanor, even though genetic factors play important roles in the pathology of schizophrenia. Indeed, no molecular-level test exists to assist diagnosis, which has limited treatment strategies. To address this serious shortcoming, we used a bioinformatics approach to identify 61 genes that are differentially expressed in schizophrenia patients compared with healthy controls. In particular, competing endogenous RNA network revealed the important role of the gene *RASD2*, which is regulated by miR-4763-3p. Indeed, analysis of blood samples confirmed that *RASD2* is downregulated in schizophrenia patients. Moreover, positron emission tomography data collected for 44 human samples identified the prefrontal and temporal lobes as potential key brain regions in schizophrenia patients. Mechanistic studies indicated that miR-4763-3p inhibits *RASD2* by base-pairing with the 3’ untranslated region of *RASD2* mRNA. Importantly, *RASD2* has been shown to interact with *β-arrestin2*, which contributes to the regulation of the DRD2-dependent *CREB* response element-binding protein pathway in the dopamine system. Finally, results obtained with a mouse model of schizophrenia revealed that inhibition of miR-4763-3p function alleviated anxiety symptoms and improved memory. The dopamine transporters in the striatal regions were significantly reduced in schizophrenia model mice as compared with wild-type mice, suggesting that inhibition of miR-4763-3p can lessen the symptoms of schizophrenia. Our findings demonstrate that miR-4763-3p may target *RASD2* mRNA and thus may serve as a potential biomarker and therapeutic target for schizophrenia, providing a theoretical foundation for further studies of the molecular basis of this disease.

Schizophrenia is a severe mental illness involving a variety of psychological disorders with both positive symptoms (fantasies, as well as speech and behavioral disorders) and negative symptoms (exaggerated mood swings) [1], and such cognitive deficits are characteristic of schizophrenia [2]. Schizophrenia affects approximately 1% of the population worldwide, with serious negative effects on quality of life. The disease is most often diagnosed during adolescence or early adulthood [3]. The cause of schizophrenia remains unclear. At the post-transcriptional level, microRNAs (miRNAs) play a vital role in brain development and the pathophysiology of many mental illnesses [4]. For example, a study of miRNAs in the dorsal lateral prefrontal cortex of patients with schizophrenia revealed the temporal dynamics of miRNA expression in this region during different perinatal and young-adult periods[5]. That study suggested a possible connection between schizophrenia and the dysregulation of certain miRNAs [6]. Nevertheless, the molecular mechanism underlying the pathogenesis of schizophrenia remains unclear, which has limited the development of novel treatments.

Biomarkers are biochemical indicators that can be used to monitor pathophysiological changes in cells, tissues, and organs at the molecular level. Owing to their specificity of expression in a particular physiological context as well as a high sensitivity of detection, biomarkers can inform prognosis and be used for auxiliary diagnosis. Numerous technologies have been employed in the search for biomarkers specific for schizophrenia. miRNAs are quite stable, and each miRNA regulates the expression of one or more specific mRNA; consequently, recent research has suggested that miRNAs may be attractive candidate biomarkers for schizophrenia [7]. In particular, miRNAs in whole blood or specific blood components are candidates for improving the diagnosis of several diseases, including life-threatening pathology [8], certain blood-borne miRNAs may help predict the pathogenesis of schizophrenia [9].

The dopamine system is vital for brain function and helps regulate sports behavior, cognition and emotion [10]. Some studies have revealed enhanced dopamine synthesis in the striatum of patients with schizophrenia, as well as an increased release of dopamine [11]. Dopamine receptors can be divided into two major families: D1 and D2. Traditionally, dopamine-associated neuro-transmission has been thought to occur through five different receptors, including those of the D2 family, that transduce dopaminergic signals [12]. The D2 dopamine receptors are the most widely studied family for the treatment of schizophrenia and therefore may be therapeutic targets. Brain imaging studies have revealed significant regulation of D2 receptor expression in the brain of schizophrenia patients [13]. Consistent with this report, drugs approved to treat schizophrenia block D2 receptor signaling [14]. In addition to the dopamine receptors, serotonin (5-hydroxytryptamine, 5-HT) receptors have attracted widespread attention as potential therapeutic targets for cognitive disorders including schizophrenia [15].

The Ras superfamily small GTPase named RASD Family Member 2 (RASD2) is enriched in the striatum. RASD2 is one of the activators of mechanistic target of rapamycin 1 (mTOR1) which in turn plays a role in myelination as well as axon growth and regeneration. We previously reported that RASD2 is significantly downregulated in schizophrenia patients compared with healthy control subjects [16]. Therefore, we can infer that RASD2 may indirectly affect signal transduction in the central nervous system—and hence the symptoms of schizophrenia—through endocrine function.

In the present study, we applied a bioinformatics analysis to identify differentially expressed genes (DEGs) between patients with schizophrenia and healthy individuals. We also analyzed human blood samples to determine whether certain miRNAs could be potential biomarkers for schizophrenia. The results revealed a significant downregulation of *RASD2* and upregulation of the miRNA miR-4763-3p in blood samples from schizophrenia patients. Moreover, inhibition of miR-4763-3p resulted in significant improvement of schizophrenia symptoms as well as enhance learning memory and recovery from anxiety in a mouse model of schizophrenia (SCZ mice). Therefore, we explored the role of *RASD2* and its regulation by miR-4763-3p in dopamine-associated signaling pathways[15].

## MATERIALS AND METHODS

### Animals

C57BL/6 (wild-type, WT) mice were reared at a constant temperature (22 ± 1°C) and maintained on a 12/12 h light/dark cycle, with food and water provided ad libitum. All animals were treated in accordance with the International Guidelines for Animal Research. The study design was approved by the Animal Ethics Committee of Shanghai University.

### Positron emission tomography (PET)

According to normal operating procedures, PET was performed on mice using Inveon PET/CT System (Siemens Medical Solutions, Knoxville, KY, USA) at the PET Center of Huashan Hospital, Fudan University. Briefly, mice were anesthetized using isoflurane, and then 0.5 mCi 2'-methoxyphenyl-(N-2'-pyridinyl)-p-^18^F-fluoro-propyl-benzamidoethylpiperazine (^18^F-labeled MPPF),^18^F-radiolabeled N-(3-fluoropropyl) 2β-carboxymethoxy-3β-(4-iodophenyl) nortropane (^18^F-FP-CIT) was injected through the tail vein. After 50 min, PET was carried out for a total of 10 min. Mice were anesthetized using isoflurane.

### Identification of DEGs and functional annotation

To identify the DEGs between schizophrenia patients and healthy individuals, previously reported raw RNA-Seq data were obtained [17] (GEO accession number GSE121376). A locally developed Perl script was used to delete low-quality bases at the 5′ and 3′ ends. HISAT2 (version 2.0.5) was used to map reads to the human transcriptome (GRCm38.p5). Read counts were generated using BED Tools [18], and expression values were calculated using the RPKM (reads per kilobase per million mapped reads) method. DEGs were identified using a fold-change threshold of >1.50 or <0.67, and p-values were obtained from a t-test; p<0.05 was considered to reflect a statistically significant difference between values. DAVID 6.8 was used for functional enrichment analysis of schizophrenia-related genes [19].

### Construction of a competing endogenous RNA (ceRNA) network

miRNAs were collected from miRbase [20], and mRNA and long noncoding RNA (lncRNA) sequences were obtained from NCBI (GRCm38.p5). All differentially expressed mRNAs and lncRNAs were used for target identification. The miRNA-mRNA and miRNA-lncRNA regulatory relationships were determined using RNAhybrid (setting the seed region from coordinates 1 to 6, 2 to 7, 3 to 8, 4 to 9, and 5 to 10; minimum free energy < -25 kcal/mol, p < 0.01) and BLASTN (word size of 10 and e-value of 1000, removing the miRNA from the forward alignment and miRNA position of alignment greater than 6), and the intersection was obtained. The ceRNA network was visualized using Cytoscape 3.5.1 [21].

### Behavior analysis

The forced swimming test was performed in a transparent glass cylinder of diameter 20 cm and height 40 cm. The test was performed for 6 min; during the first 2 min, the mice were adapted to the environment, and then immobility time was recorded during the last 4 min of the test. After each test, the mouse was dried, and the water in the cylinder was changed.

The open-field test was carried out with an open-field box (40 cm × 40 cm). Noldus software was used to assess the time spent in the central zone as well as the total distance traveled for 10 min in the open field. The distance each mouse moved was inferred as exercise ability, and the time spent in the central zone was used to assess anxiety behavior. Between tests, the apparatus was cleaned with aqueous 75% ethanol to mask the scent of previously tested animals.

Novel object recognition (commonly known as NOR) was performed in an open-field box (40 cm × 40 cm). Mice were allowed to acclimate to the testing room for 48 h. On the third day, two identical objects were placed in the box, and the mouse was allowed to freely explore for 10 min, and then one of the objects was replaced followed by a second 10-min free exploration. Noldus software was used to analyze the total distance moved, residence time, number of entries, and average speed of the experimental animals entering the area around the two objects. Between tests, the apparatus was cleaned with aqueous 75% ethanol.

### Brain imaging

A cohort of 44 individuals (18 with schizophrenia and 26 healthy controls) was collected from Huashan Hospital, Shanghai, China. All Digital Imaging and Communications in Medicine (commonly known as DICOM) data were converted into NIfTI-formatted files using DCM2NII (http://people.cas.sc.edu/rorden/mricron/index.html). Two steps were used to establish the boundaries of the brain for analysis. In the preprocessing stage, statistical parametric mapping (SPM12, www.fil.ion.ucl.ac.uk/spm) was implemented in MATLAB R2016a; every image was normalized to a standard brain space by the ICBM East Asian template, and the low-frequency background noise was simultaneously removed. Next, an isotropic Gaussian smoothing kernel with a full width at half maximum (FWHM) value of 10 × 10 × 10 mm³ was applied. Regions of interest were compared between the schizophrenia and healthy-subject groups using a two-sample Student’s t-test. Regions could be localized by setting the peak threshold to p < 0.01 and applying an error correction with a threshold of 20 voxels. Talairach Client enabled the creation of individual and batch labels of the regions of interest.

### RNA extraction from mouse brain and quantitative real-time PCR (qPCR)

The prefrontal lobe of mice was dissected quickly in phosphate-buffered saline (PBS) on ice and placed in RNA extraction solution (Promega, Madison, WI, USA) and extracted according to the manufacturer’s protocol. The final reaction volume contained 2 µg total RNA, 4 μL of 5× RT Master Mix (TaKaRa, Kusatsu, Japan), and RNase-free water (up to 20 μL). cDNA was synthesized at 25°C for 5 min, 37°C for 30 min, 85°C for 10 s, and 12°C for 10 min. To quantify the target genes, qPCR was performed using qPCR SYBR Green Master Mix (Yeasen, Shanghai, China). Each reaction contained 1 μL cDNA sample (100-200 ng/μL), 10 μL qPCR SYBR Green Master Mix, 0.8 μL (10 µM) designated primers, and RNase-free water (up to 20 μL). The PCR conditions were as follows: 95°C for 30 s, 55°C for 20 s, and 72°C for 30 s, with 40 thermal cycles. The mRNA normalized to that of GAPDH.

**Figure 1.**
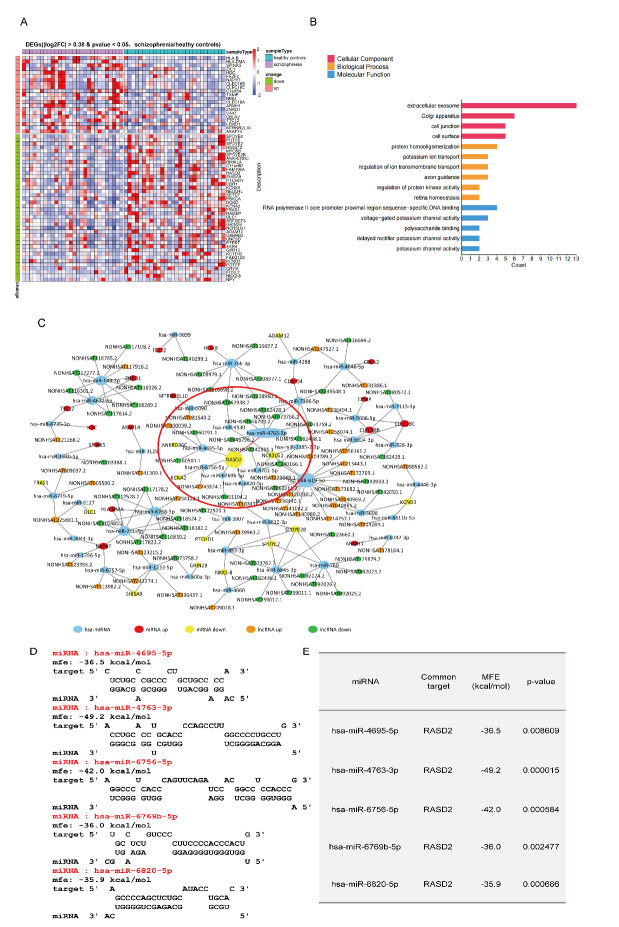
***RASD2* as the hub gene among the DEGs between patients with schizophrenia and healthy controls**. (**A**) Heatmap showing upregulation (red) and downregulation (blue) of DEGs between schizophrenia patients and healthy controls. The thresholds for DEG identification were as follows: | log2FC | > 0.38 and p < 0.05, schizophrenia vs. healthy controls. (**B**) Gene Ontology functional enrichment analyses for cellular components (red), biological processes (yellow), and molecular functions (blue). (**C**) ceRNA network for DEGs (miRNA-mRNA and miRNA-lncRNA) for upregulated mRNAs (red nodes), downregulated mRNAs (yellow nodes), upregulated lncRNAs (orange nodes), downregulated lncRNAs (green nodes), interactions between two nodes (gray lines), and *RASD2*-miR-4763-3p, which was the main focus of subsequent investigations (red line). (**D**) miRNA-mRNA interactions predicted by RNAhybrid for *RASD2* mRNA. (**E**) miRNA-mRNA interactions showing values for *RASD2*, the mRNA for which is regulated by at least five different miRNAs. MFE: minimum free energy.

### RNA extraction from blood and qPCR

Blood was collected in tubes from 20 patients with schizophrenia and 6 healthy controls, and serum was obtained after coagulation and placed in RNA extraction solution (Yeasen). RNA was extracted according to the manufacturer’s protocol. The final reaction volume contained 1 µg total RNA, 2 μL of 5× Reverse Transcription Buffer, RT Master Mix (RiboBio, Guangzhou, China), and miRNA reverse-transcription primer (1 μL). cDNA was synthesized at 42°C for 60 min and 70°C for 10 min. To quantify the target genes, qPCR was performed using qPCR SYBR Green Master Mix (RiboBio). Each reaction contained 2 μL reverse-transcription product, 10 μL qPCR SYBR Green Master Mix, 0.8 μL (200 nM) designated primers, and RNase-free water (up to 20 μL). The PCR conditions were as follows: 95°C for 10 min, followed by 40 cycles of 95°C for 2 s, 60°C for 20 s, and 70°C for 10 s. The mRNA level in each sample normalized to that of U6.

### Western blotting

Total cellular protein was extracted using a cell lysis buffer (Beyotime, China) according to the manufacturer’s protocol. Each homogenate was centrifuged at 12,000 rpm for 30 min at 4°C, and the supernatant was collected for measuring soluble protein concentration using the BCA-100 protein assay kit. An equal mass of protein (20 μg) from each sample was boiled for 10 min in 5× SDS-PAGE loading buffer and then subjected to SDS-PAGE (12% polyacrylamide gel). The separated proteins were transferred onto a nitrocellulose filter membrane, which was blocked with 5% bovine serum albumin (Solarbio, Beijing, China) for 60 min at room temperature, then incubated overnight at 4°C with a primary antibody. The next day, the membrane was incubated with a secondary antibody at room temperature for 1 h. Visualization and quantification of immune-positive bands were carried out using Odyssey scanner and associated software (LI-COR Biosciences, USA). The relative protein level was normalized to that of GAPDH from the same lane.

### A mouse model of schizophrenia (SCZ mice)

The N-methyl-d-aspartate (NMDA)-type glutamate receptor antagonist MK-801 (Sigma-Aldrich, St. Louis, MO, USA) was dissolved in physiological saline and injected into the abdominal cavity of mice for 7 days (0.6 mg/kg, once daily)[22]. As a control group, mice were injected with the same amount of saline. PET and qPCR experiments were performed within 1 week after injection.

### Stereotactic injection

Mice were divided into six groups (n = 5 per group). Targeted injection into the hippocampus was performed with PBS, the agomir, antagomir of miR-4763-3p, along with a negative control ((NC: sequence-scrambled miRNA and control (a blank control without treatment)). The groups of mice and treatments were as follows: (a) WT + PBS, (b) SCZ + miR-4763-3p antagomir, (c) SCZ + antagomir NC, (d) SCZ + miR-4763-3p agomir, (e) SCZ + agomir NC. First, we made a longitudinal incision to expose the bregma, set this point as zero, and determined the CA1 region of the hippocampus from this point (anterior-posterior: +2.0 mm; medial-lateral: ±0.3 mm; dorsal-ventral: +1.9 mm). Each mouse was injected with 2 μL for 10 min and retained for 10 min before the needle was slowly retracted. After 7 days with free access to normal food, the mice were subjected to behavioral testing.

### Statistical analysis

All data were analyzed using GraphPad Prism, and the results are presented as the mean ± SEM. Differences between two groups were assessed using the unpaired two-tailed Student’s t test. P-values of <0.05 indicated statistical significance.

## RESULTS

### RASD2 is the hub gene among the DEGs between schizophrenia patients and healthy controls

To determine molecular differences between schizophrenia patients and healthy controls, we analyzed RNA sequencing results (bulk) obtained with human cortical interneurons derived from induced pluripotent stem cells. Analysis of raw RNA-Seq data downloaded from the GEO database (www.ncbi.nlm.nih.gov/geo/; accession number GSE121376) [23] identified 61 DEGs between 28 patients with schizophrenia and 28 healthy controls. Among these 61 DEGs, 21 were upregulated and 40 were downregulated in most schizophrenia patients (Fig. 1A, Supplementary Fig. 1A). A previous study found that mutations in human leukocyte antigen B (HLA-B) in the prefrontal lobe may affect dopamine signaling in the central nervous system and indirectly influence the development of schizophrenia [16]. In addition, dysfunction of glutamatergic signaling in the brain may be involved in the pathophysiology of schizophrenia, and myosin Vb (MYO5B) contributes to glutamate receptor 1(GluR1) signaling, suggesting a link between glutamatergic signaling and schizophrenia [24]. Therefore, the DEGs identified in our analysis are related to schizophrenia.

**Figure 2.**
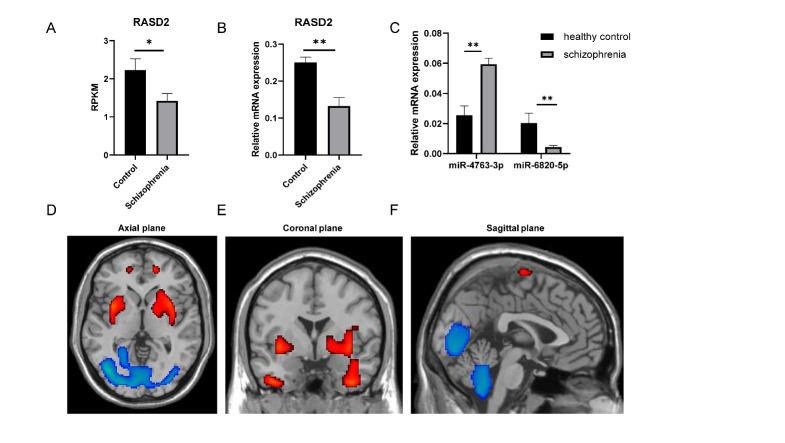
**Blood-sample analysis of schizophrenia patients as well as PET scans of pathological brain areas**. (**A-C**) Blood-sample analysis showing (**A**) RPKM values for *RASD2*, (**B**) *RASD2* expression in blood samples, and (**C**) normalized relative expression of miR-4763-3p and miR-6820-5p in the schizophrenia group and healthy control group. Data represent the mean ± SEM, n ≥ 3;^*^p < 0.05,^**^p < 0.01. (**D**) PET scans showing brain areas with enhanced (red) or reduced (blue) prevalence of the 5-HT receptor.

To further explore the relationship between the functions of the 61 DEGs and schizophrenia, we performed Gene Ontology enrichment analysis for functional classification using DAVID (Error! Hyperlink reference not valid.). Gene Ontology analysis revealed that most of the DEGs were enriched in the category’s extracellular exosomes, axon guidance, and RNA polymerase II sequence-specific DNA binding. This indicated that the 61 DEGs may be involved in signal transduction mediated by both intracellular and extracellular substances (Fig. 1B). Furthermore, the inferred molecular mechanisms underlying these schizophrenia-related DEGs revealed that miRNAs (the intermediate node in the ceRNA network) regulate the expression of certain of these DEGs as well as certain lncRNAs (Fig. 1C). This showed that aberrant expression of miRNAs related to one or more of these DEGs may contribute to the pathogenesis of schizophrenia. For instance, miR-619-5p (light blue nodes inFig. 1C) regulates the expression of many lncRNAs and mRNAs. Notably, *RASD2* (the central yellow node/hub in the network ofFig. 1C) is regulated by a variety of miRNAs, including miR-4763-3p. Moreover, post-mortem analysis of the prefrontal lobe of schizophrenia patients revealed downregulated expression of *RASD2* [25]. These analyses suggested that one or more miRNAs underlie the observed downregulation of *RASD2* in schizophrenia patients. Consequently, miRNAs may be involved in the dysregulation of certain DEGs in schizophrenia.

Furthermore, 43 interactions were detected between miRNAs and the 61 DEGs (Fig. 1C), and images were constructed using RNAhybrid and BLASTN for validation (Fig. 1D and E, Supplementary Fig. 1B). These results facilitated the location of optimal target sites according to the binding free energy between the miRNAs and their target genes. Based on the RNAhybrid analysis, the binding sites of *RASD2* with miRNAs (Fig. 1D) revealed that *RASD2* mRNA level potentially could be regulated by miR-4763-3p, miR-6769b-5p, miR-6820-5p, miR-6756-5p, and miR-4695-5p. Notably, the binding between miR-4763-3p and *RASD2* mRNA yielded the lowest free energy value among all the miRNAs (Fig. 1E). Therefore, miR-4763-3p is the best target for *RASD2* binding.

**Figure 3.**
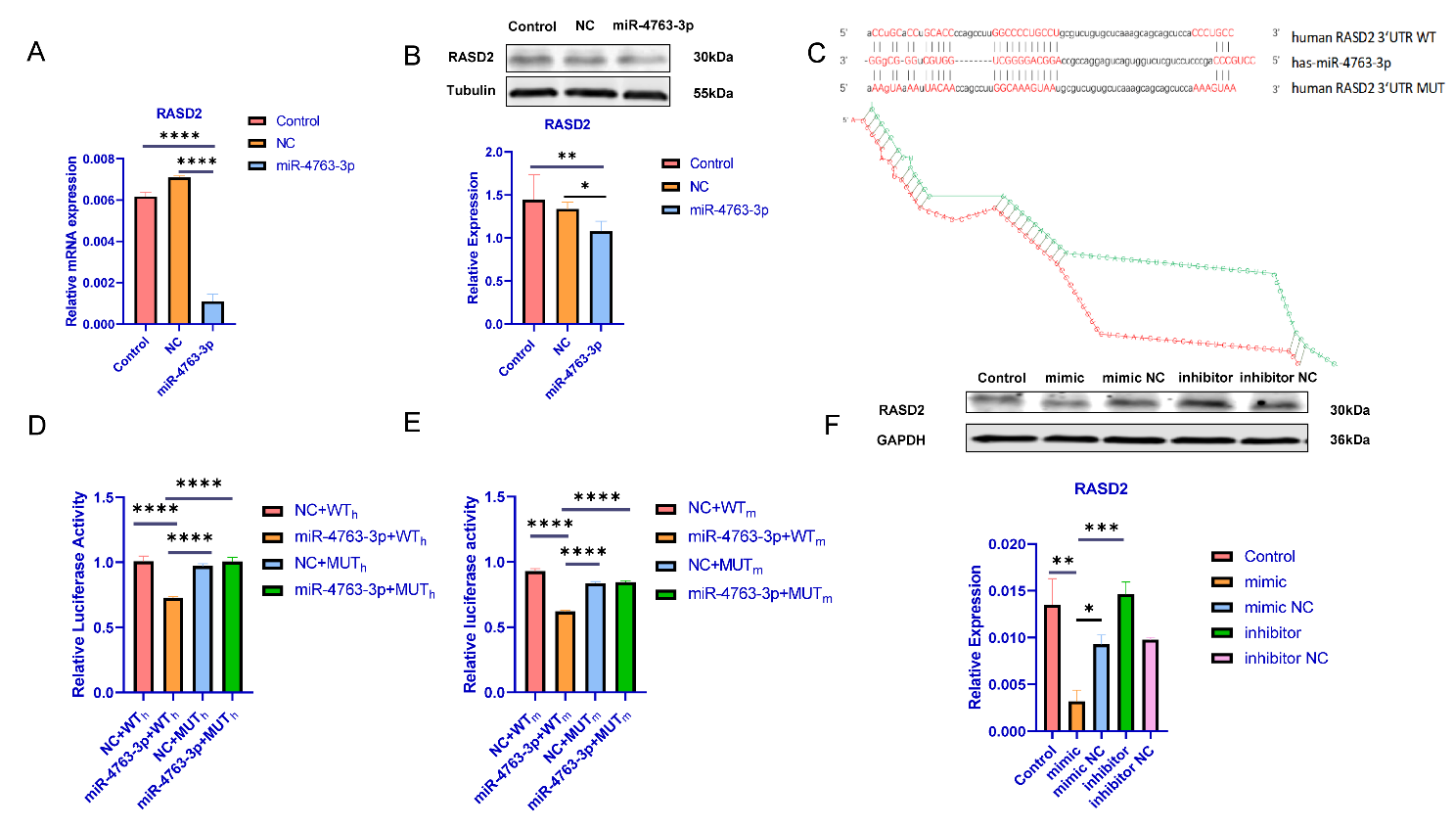
***RASD2* as a direct target of miR-4763-3p**. (**A**) *RASD2* mRNA levels in 293T cells transfected with a miR-4763-3p mimic. (**B**) RASD2 protein levels in N2A cells transfected with a miR-4763-3p mimic or the corresponding NC, as assessed with western blotting. (**C**) Predicted binding sites for miR-4763-3p in *RASD2* mRNA—both wild-type (WT) and with a mutated ( *RASD2*-MUT) 3’UTR. (**D**) Luciferase reporter assays in 293T cells co-transfected with WT or *RASD2*-MUT 3’UTR and miR-4763-3p mimic or mimic NC. (**E**) Luciferase reporter assays in N2A cells co-transfected with WT or *RASD2*-MUT and miR-4763-3p mimic or mimic NC. (**F**) RASD2 protein levels in 293T cells transfected with a miR-4763-3p mimic, miR-4763-3p inhibitor, or the corresponding NC, as assessed with western blotting. Data represent the mean ± SEM, n ≥ 3; *p < 0.05, **p < 0.01, ***p < 0.001, ****p < 0.0001.

### Blood-sample analysis of schizophrenia patients as well PET of pathological brain areas

Next, we used the RPKM values for miRNAs to verify the interactions between miRNA and *RASD2* mRNA (Fig. 2A). For the purpose here, the RPKM values reflected the strength of the correlation between expression of miRNAs and their target genes. The RPKM values for *RASD2* in the schizophrenia group were significantly altered compared with those in the healthy controls group. Subsequently, qPCR analysis of blood samples revealed that *RASD2* was significantly downregulated in the schizophrenia group compared with the control group (Fig. 2B). The qPCR analysis also revealed that, among the many *RASD2*-related miRNAs we analyzed, miR-4763-3p was significantly upregulated [26] and miR-6820b-5p was significantly downregulated (Fig. 2C). Few studies have been conducted on miR-6820b-5p, and thus interpretation of its downregulation in this context will require further investigation. Nonetheless, these results suggested that miR-4763-3p may be a diagnostic target for schizophrenia and therefore warranted further exploration.

To elucidate the key brain areas involved in schizophrenia, we analyzed PET data acquired with schizophrenia patients. The neurotransmitter 5-HT itself can induce the release of certain other neurotransmitters in the prefrontal cortex, including dopamine [27,28]. In fact, as early as 1954, Wolley *et al*. hypothesized that schizophrenia may be related to disorders of 5-HT metabolism [29]. PET was used to specifically detect 5-HT receptors [30] in blood samples from 26 healthy controls and 18 schizophrenia patients who had been injected with^18^F-MPPF, a highly selective 5-HT antagonist [31], and the PET data were analyzed with MATLAB-R2016a. The results revealed upregulated expression of 5-HT receptors in the frontal, temporal and sub-lobal areas in the brain of schizophrenia patients whereas expression was downregulated primarily in the occipital lobe, posterior lobe and left temporal lobe (Fig. 2D-F).

**Figure 4.**
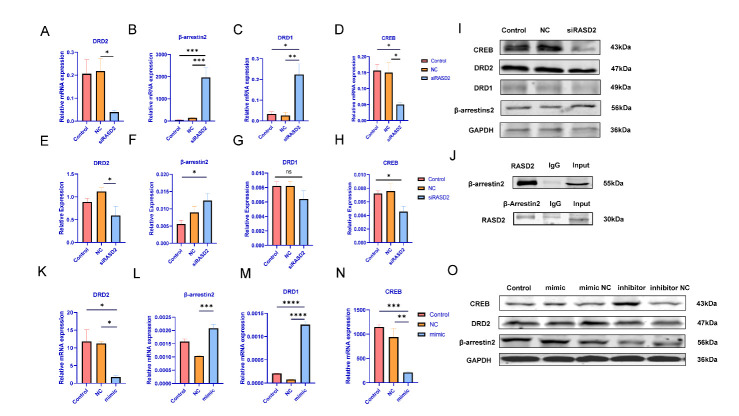
**miR-4763-3p is involved in the D1- and D2-dependent signaling pathways by regulating the target gene *RASD2***. (**A-D**) Expression of mRNAs encoding *DRD2, β-arrestin2, DRD1*, and *CREB* in 293T cells transfected with si *RASD2* and the corresponding negative control (NC) as assessed with qPCR, (**E-I**) and expression of the proteins as assessed with western blotting. (**J**) A co-immunoprecipitation assay was used to detect the association between RASD2 and β-arrestin2. (**K-N**) Expression of mRNAs encoding *DRD2, β-arrestin2, DRD1, CREB* in 293T cells transfected with a miR-4763-3p mimic, the corresponding NC, and control as assessed with qPCR, and (**O**) expression of the proteins CREB, DRD2 and β-arrestin2 as assessed with Western blotting. Data represent the mean ± SEM, n ≥ 3; *p < 0.05, **p < 0.01, ***p < 0.001, ****p < 0.0001.

### miR-4763-3p targeting regulates RASD2

As shown inFigure 1E, as mentioned earlier, the minimum free-energy values (Fig. 1E) revealed clear interactions between miRNAs and target genes. We focused on miR-4763-3p in subsequent studies because it had the lowest free binding energy with *RASD2*. Moreover, further analysis of blood samples from schizophrenia patients verified that *RASD2* expression was downregulated and that of miR-4763-3p was abnormally upregulated. On this basis, we conducted qPCR and western blotting with the blood samples to determine whether miR-4763-3p could regulate the physiological effects of *RASD2*. For this purpose, miR-4763-3p was overexpressed via transient transfection of cells with miR-4763-3p mimic, and the level of RASD2 protein was analyzed. Besides, for this purpose, miR-4763-3p was overexpressed via transient transfection of Neuro2a (N2A) cells and human embryonic kidney cells expressing SV40 T-antigen (293T) with miR-4763-3p mimic, and the level of RASD2 protein was analyzed. Overexpression of miR-4763-3p significantly decreased the relative levels of both RASD2 mRNA and protein (Fig. 3A and B).

**Figure 5.**
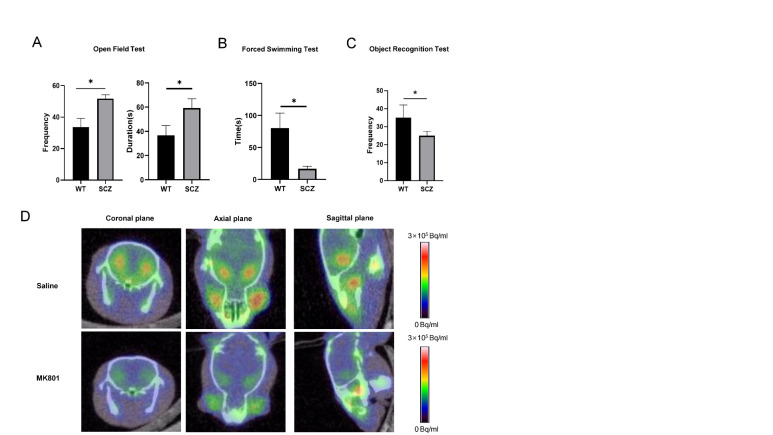
**Development of a mouse model of schizophrenia (SCZ mice)**. (**A**) Exercise ability and adaptability to a new environment were assessed using the open-field test;(B) anxiety level was assessed using the forced swimmingtest (**C**) and preference for a novel object was assessed using the novel object recognition test for mice injected with normal saline (control) and the glutamate receptor antagonist MK-801. (**D**)^18^F-FP-CIT PET images of the mouse brain showing dopamine receptors (green). Data represent the mean ± SEM, n ≥ 3; *p < 0.05.

A luciferase assay was used to further determine whether *RASD2* mRNA is a target of miR-4763-3p. Results of a bioinformatics analysis performed with TargetScan predicted that the seed sequences of miR-4763-3p are complementary to nucleotides 1778-1784 and 1814-1820 of the 3’ untranslated region (3’UTR) of human *RASD2* mRNA (Fig. 3C). 293T cells were co-transfected with plasmids encoding *RASD2*-WT mRNA and *RASD2*-MUT (mutated 3'UTR) along with a miR-4763-3p mimic or NC. The miR-4763-3p mimic significantly decreased the luciferase activity of the *RASD2*-WT transfected cells compared with the NC and *RASD2*-MUT groups, implying that miR-4763-3p markedly downregulated the reporter activity (Fig. 3D). Similar results were obtained with N2A cells (Fig. 3E), indicating that miR-4763-3p can target and regulate *RASD2* mRNA irrespective of species differences (i.e., between human and mouse). Western blotting was used to further evaluate the effects of miR-4763-3p on RASD2 protein expression in 293T cells transfected with a miR-4763-3p inhibitor, miR-4763-3p mimic, or NC. The miR-4763-3p mimic caused a significant decrease in *RASD2* mRNA level, but the effect was markedly reversed upon subsequent treatment with the miR-4763-3p inhibitor (Fig. 3F). These results demonstrated that *RASD2* mRNA might be a direct target of miR-4763-3p.

**Figure 6.**
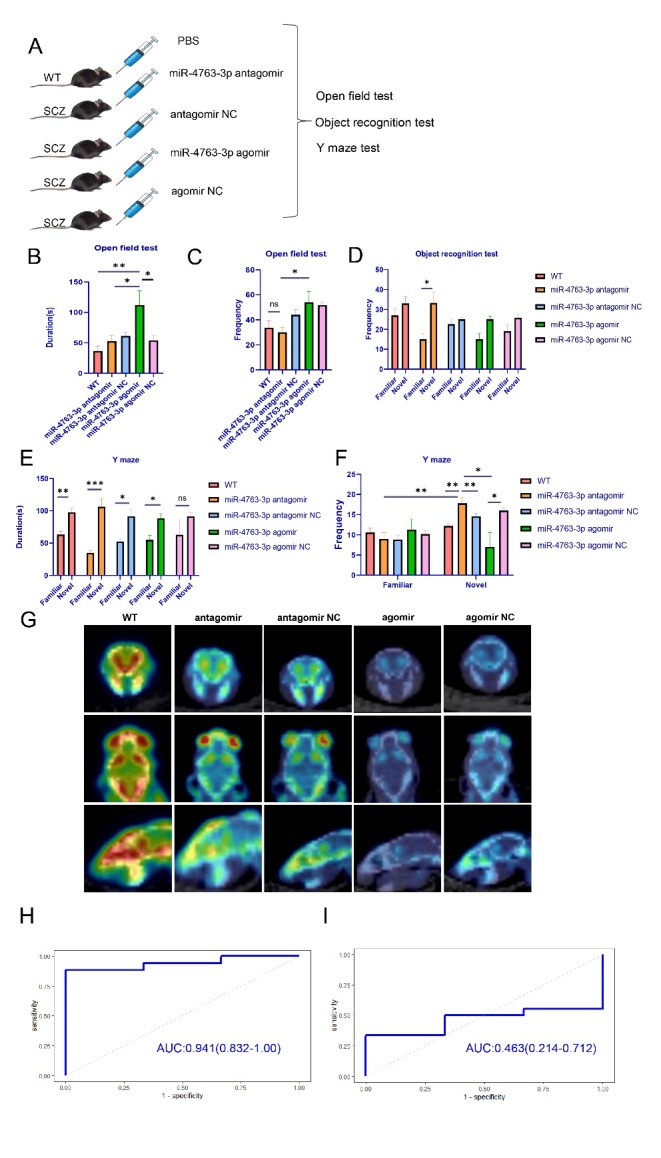
**miR-4763-3p antagomir relieves the symptoms of schizophrenia**. (**A**) Schematic illustration of the stereotactic injection of the agomir antagomir into the brain of WT or SCZ mice. Two-month-old mice were divided into five groups: WT, miR-4763-3p agomir, miR-4763-3p agomir negative control (NC), miR-4763-3p antagomir, and miR-4763-3p antagomir NC. (B, C) Exercise ability and adaptability to a new environment were assessed using the open-field test to measure duration (**B**) and frequency (**C**). (**D**) Preference for a novel object was assessed using the object recognition test. (E, F) Spatial memory ability was assessed using the Y-maze test to measure duration (**E**) and frequency (**F**). (**G**) Representative^18^F-radiolabeled N-(3-fluoropropyl) 2β-carboxymethoxy-3β-(4-iodophenyl) nortropane (^18^F-FP-CIT) PET computed tomography images of the brain of SCZ mice treated with a miR-4763-3p agomir, miR-4763-3p agomir NC, miR-4763-3p antagomir, or miR-4763-3p antagomir NC; the green signal indicates dopamine transporters. (H, I) Receiver operating characteristic analysis of miR-4763-3p from schizophrenia patients and healthy controls (AUC: area under the curve). Data represent the mean ± SEM, n ≥ 3; *p < 0.05, **p < 0.01, ***p < 0.001.

### miR-4763-3p is involved in both the D1- and D2-dependent signaling pathways by regulating translation of RASD2 mRNA.

*RASD2* is involved in dopamine signaling in schizophrenia[16]. For instance, DRD2-dependent cAMP response element-binding protein (CREB) signaling is mediated by the inhibitory dopamine receptor DRD2, whereas protein kinase A signaling is mediated by the regulation of the excitatory dopamine receptor DRD1. Therefore, we investigated whether miR-4763-3p is involved in these pathways via its regulation of *RASD2* mRNA level. Toward this end, we first constructed an expression vector for a short interfering RNA (siRNA) targeting *RASD2* mRNA, and 293T cells were co-transfected with this vector along with a negative control (NC: sequence-scrambled siRNA and control (a blank control without treatment). Indeed, siRNA-mediated *RASD2* silencing in 293T cells led to a decrease in both the mRNA and protein levels of DRD2 and CREB in the DRD2-mediated signaling pathway. We found that in the DRD2-mediated signaling pathway, DRD2 and CREB mRNA and protein levels in 293T cells were decreased, whereas β-arrestin2 mRNA and protein levels increased significantly (Fig. 4A-I). Previous studies have suggested that *β-arrestin2* may interact with RASD2 to regulate the CREB pathway, as demonstrated by immunoprecipitation with an antibody against RASD2 followed by western blotting for β-arrestin2 [32]. Our results indicated that RASD2 was associated with β-arrestin2 in vivo but not with the IgG control (Fig. 4J). This result was confirmed via reciprocal immunoprecipitation with anti-β-arrestin2 and western blotting with anti-RASD2. Moreover, a co-immunoprecipitation experiment demonstrated that RASD2 interacted with β-arrestin2, which could potentially regulate CREB signaling (Fig. 4J). These results clearly demonstrated that RASD2 positively regulates D2R-dependent signaling by interacting with β-arrestin2, whereas RASD2 negatively regulates D1R-dependent signaling.

To explore whether miR-4763-3p, via its role in regulating *RASD2* mRNA level, is involved in the D1- and D2-mediated signaling pathways, 293T cells were transfected with a miR-4763-3p mimic or NC. Overexpression of miR-4763-3p significantly decreased the mRNA levels of *DRD2* and *CREB*, whereas the levels for *DRD1* and *β-arrestin2* mRNAs were significantly increased. (Fig. 4K-N). To confirm these results, western blotting was carried out with extracts of 293T cells transfected with a miR-4763-3p inhibitor, miR-4763-3p mimic, or NC. Indeed, the levels of proteins involved in DRD2 signaling underwent distinct alterations. For example, DRD2 level decreased significantly in the miR-4763-3p mimic group relative to the mimic NC group, whereas expression of the inhibitor resulted in slight recovery of the DRD2 level. In addition, the level of β-arrestin2 was markedly upregulated in the mimic group yet downregulated in the inhibitor group (Fig. 4O). These results were confirmed in N2A cells (Supplementary Fig. 2). Overall, these results confirmed that miR-4763-3p—through its role in modulating the level of *RASD2* mRNA—is involved in D2-mediated signaling.

### Establishment of a mouse model of schizophrenia

In order to explore the regulatory mechanism of previous results. We established SCZ mice via intraperitoneal injection of the NMDA receptor antagonist MK-801 [22]. Notably, significant differences were found in behavioral analysis, compared with saline-injected control mice, administration of MK-801 resulted in obvious anxiety-like behaviors of mice in open-field test (Fig. 5A) and decreased mobility in the forced swim test (Fig. 5B). In addition, the memory ability of MK-801-injected mice was significantly reduced in the novel object recognition test (Fig. 5C). These results were consistent with the characteristics of schizophrenia. In fact, schizophrenia is often accompanied by abnormal expression of dopamine receptors [33]. Using PET to detect the expression level of dopamine receptors in brain regions can assess the occurrence of schizophrenia. We injected^18^F-FP-CIT via the tail vein and detected dopamine transporter expression [1,34]. Our data showed decreased expression in the striatum of MK-801 group (Fig. 5D), further validating the schizophrenia-like attributes of SCZ mice.

### A miR-4763-3p antagomir relieves the symptoms of schizophrenia in SCZ mice

Because miR-4763-3p downregulated *RASD2* mRNA level in cells, we further investigated whether it is also involved in dopamine-related signaling. For this purpose, we used 2-month-old mice to generate a schizophrenia model (SCZ mice) involving stereotactic injection of the agomir or antagomir of miR-4763-3p (along with the PBS control and NC groups) into the hippocampal area of the mouse brain (Fig. 6A). Behavioral experiments were carried out one week later, which revealed notable changes in behavior. For example, in the open-field test (to assess anxiety), the SCZ mice treated with miR-4763-3p agomir manifested a higher anxiety level than control mice (Fig. 6B and C). In addition, the object recognition test (to assess spatial learning) revealed that SCZ mice spent less time dwelling on novel objects compared with control mice. The SCZ mice that received the miR-4763-3p antagomir, miR-4763-3p agomir, NC, or PBS spent an almost equivalent amount of time exploring in the original object. For the object recognition test, the control mice or SCZ mice that had been treated with the miR-4763-3p antagomir found the novel object more frequently than the miR-4763-3p agomir mice or NC mice (Fig. 6D). Finally, Y-maze was used to evaluate memory improvement. The memory ability of SCZ mice treated with the miR-4763-3p agomir was significantly reduced compared with the NC mice, whereas the memory of the miR-4763-3p antagomir mice was remarkably improved. These results indicated that miR-4763-3p overexpression can lead to more severe schizophreniform symptoms. In contrast, inhibition of miR-4763-3p expression significantly improved the memory of SCZ mice (Fig. 6E and F). These results demonstrated that downregulation of miR-4763-3p rescues memory, cognition impairment, and anxiety level in SCZ mice compared with the control mice. In contrast, overexpression of miR-4763-3p causes cognitive decline, a reduction in memory ability, and anxiety-like behavior in SCZ mice in vivo.

Dopamine transporters have been associated with many neurological disorders including schizophrenia [35]. Detection of the levels of dopamine transporters by PET can be used to diagnose schizophrenia[37]. In the present study, the dopamine transporter imaging agent^18^F-FP-CIT was injected through the tail vein of SCZ mice in treatment groups, and dopamine transporter expression was detected using PET. The SCZ mice treated with the miR-4763-3p antagomir had higher dopamine transporter expression compared with the miR-4763-3p antagomir NC; the results for the miR-4763-3p agomir and the NC groups did not differ significantly from those acquired for the miR-4763-3p antagomir group (Fig. 6G). These findings supported our previous data showing that the miR-4763-3p antagomir could lessen the symptoms of schizophrenia in SCZ mice.

**Figure 7.**
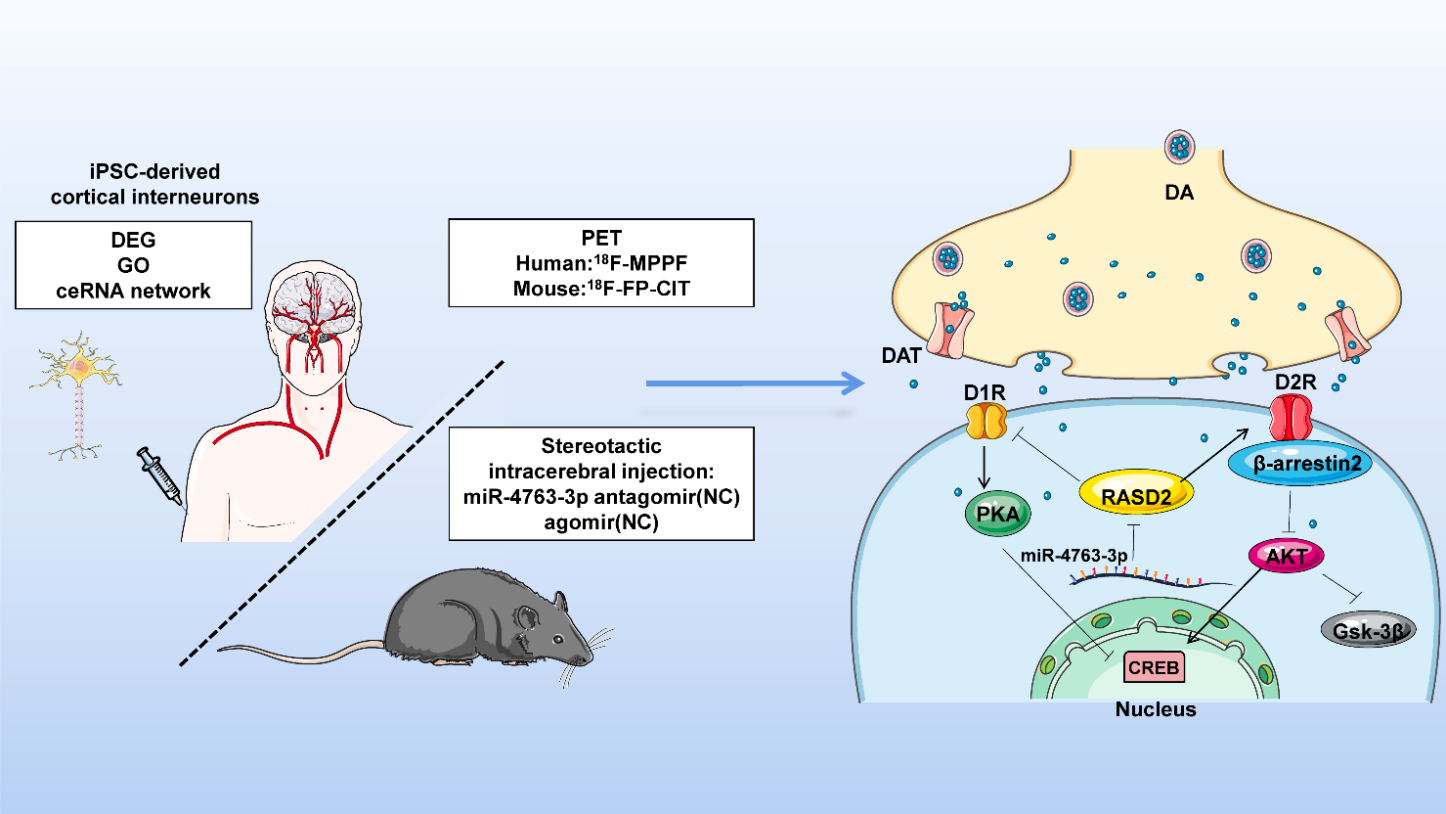
**miR-4763-3p targets *RASD2* mRNA and thus may be a potential biomarker and therapeutic target for schizophrenia**. miR-4763-3p targets *RASD2* mRNA, which is involved in the regulation of the D1- and D2-dependent CREB signaling pathways and may serve as a potential biomarker and therapeutic target for schizophrenia, providing a theoretical foundation for further studies on the molecular basis of schizophrenia. iPSC: induced pluripotent stem cells.

### Evaluation of miRNAs in plasma as novel biomarkers for schizophrenia

To further investigate the sensitivity, specificity, and area under the curve (AUC) of miR-4763-3p as a potential biomarker for schizophrenia, we performed receiver operating characteristic analysis with the schizophrenia patients before and after treatment by extracting peripheral blood to assess the value of miR-4763-3p for SCZ progress diagnosis (Fig. 6H and I). miR-4763-3p was effective in this regard for schizophrenia vs control (average AUC of 0.941, 95% confidence interval 0.834 to 1.00) (Fig. 6H). However, miR-4763-3p was ineffective for the comparison of post-treatment schizophrenia vs control (average AUC of 0.463, 95% confidence interval 0.214 to 0.712) (Fig. 6I). These results clearly indicated that miR-4763-3p could potentially serve as a biomarker for distinguishing patients with schizophrenia from healthy individuals, with both high sensitivity and specificity. Moreover, the data suggested that miRNAs in whole blood or specific blood components could serve as candidate biomarkers for improving the diagnosis of other diseases, including those with life-threatening pathologies [39].

## DISCUSSION

Functional enrichment analysis of the 61 schizophrenia-related DEGs we identified revealed that most are enriched in synaptic functioning and neurotransmitter-related signaling (Fig. 1B), and the ceRNA network indicated that *RASD2* plays a key role in schizophrenia (Fig. 1C-E). Subsequent analysis of human blood samples verified that the expression of *RASD2* was downregulated and that of miR-4763-3p, which targets *RASD2*, was upregulated in schizophrenia patients (Fig. 2A-C). In addition, PET confirmed that schizophrenia patients have altered brain regions such as the frontal, temporal, and occipital lobes (Fig. 2D-F). Luciferase assays were performed to confirm that miR-4763-3p targets *RASD2* mRNA and that miR-4763-3p decreases the cellular level of both *RASD2* mRNA and protein (Fig. 3), which was found to be involved in regulating the DRD2-dependent CREB pathway in the dopamine system (Fig. 4). To validate the aforementioned findings, we developed SCZ mice and performed behavioral tests and PET analysis (Fig. 5). After seven days of behavioral observation, the mice overexpressing miR-4763-3p were more anxious and had significantly decreased memory capacity. Contrastingly, the inhibition of miR-4763-3p significantly improved anxiety and restored learning and memory—similar to results obtained for healthy controls. Our results strongly suggest that miR-4763-3p may be a potential biomarker for schizophrenia and a likely target for the development of novel therapeutics (Fig. 7).

We also demonstrated that miR-4763-3p significantly inhibits RASD2 function and thus itself may prove efficacious for the treatment of schizophrenia. Moreover, RASD2 was mainly enriched in the striatum of both healthy subjects and schizophrenia patients. This is the most dopamine receptor-enriched brain region, mainly distributed into D1- and D2-like receptors[40]. While exploring the role of RASD2 in these D1- and D2-dependent signaling pathways, after silencing of *RASD2*, we noted upregulation of D1R and β-arrestin2 expression and downregulation of D2R and CREB expression. This positive correlation between RASD2 and D2R expression is consistent with results reported by Veronica Ghiglieri *et al*. [41]. Moreover, reduced expression of CREB negatively affects memory and promotes schizophreniform symptoms, which is also consistent with our results (Fig. 4). Thus, it is possible that pathological overexpression of miR-4763-3p in schizophrenia could downregulate *CREB* as well as the upstream genes that are involved in D1- and D2-mediated signaling. Our results suggest that β-arrestin2 interacts with RASD2 in the D2R signaling pathway. Inhibition of miR-4763-3p upregulates *RASD2*, which is involved in regulating D1- and D2-dependent signaling, thereby upregulating the activity of the cellular transcription factor CREB. These data suggest that miR-4763-3p may constitute a new epigenetic target for investigations involving SCZ mice and perhaps even the treatment of schizophrenia patients.

To validate the results from our aforementioned behavioral analysis, we found that the SCZ mice in which miR-4763-3p function was experimentally inhibited had significantly elevated levels of dopamine transporters compared with NC mice (Fig. 6G). In contrast, mice overexpressing miR-4763-3p had significantly reduced levels of dopamine transporters. Furthermore, in recent years, many miRNAs have been used for the treatment and diagnosis of disease. For example, inhibition of miR-155 expression can suppress the development of lymphoma [43], the upregulation of miR-425 can attenuate necroptosis and improve locomotor function [44], and inhibition of miR-34a can inhibit glioma progression and chemoresistance via targeting PD-L1 [45]. In our present study, for the first time, we revealed that inhibition of miR-4763-3p can alleviate the symptoms of schizophrenia. It has also been shown that miRNAs can be used as effective diagnostic markers to improve the classification of pituitary adenomas [46]. Similarly, our results demonstrate that miR-4763-3p can be a useful biomarker for schizophrenia and a potential therapeutic target for the development of novel drugs (Fig. 6G).

## Supplementary Materials

The Supplementary data can be found online at: www.aginganddisease.org/EN/10.14336/AD.2022.0103.



## References

[1] Abohamza E, Weickert T, Ali M, Moustafa AA (2020). Reward and punishment learning in schizophrenia and bipolar disorder. Behav Brain Res, 381:112298. 31622639 10.1016/j.bbr.2019.112298

[2] Miley K, Fisher M, Nahum M, Howard E, Rowlands A, Brandrett B, et al. (2020). Six month durability of targeted cognitive training supplemented with social cognition exercises in schizophrenia. Schizophr Res Cogn, 20:100171. 31908976 10.1016/j.scog.2019.100171 PMC6938953

[3] Hu TM, Ping LY, Hsu SH, Tsai HY, Cheng MC (2018). Mutation analysis of the WNT7A gene in patients with schizophrenia. Psychiatry Res, 265:246-248. 29763843 10.1016/j.psychres.2018.04.057

[4] Hunsberger JG, Austin DR, Chen G, Manji HK (2009). MicroRNAs in mental health: from biological underpinnings to potential therapies. Neuromolecular Med, 11:173-182. 19544012 10.1007/s12017-009-8070-5 PMC2754593

[5] Hu Z, Gao S, Lindberg D, Panja D, Wakabayashi Y, Li K, et al. (2019). Temporal dynamics of miRNAs in human DLPFC and its association with miRNA dysregulation in schizophrenia. Transl Psychiatry, 9:196. 31431609 10.1038/s41398-019-0538-y PMC6702224

[6] Du X, Choa FS, Chiappelli J, Wisner KM, Wittenberg G, Adhikari B, et al. (2019). Aberrant Middle Prefrontal-Motor Cortex Connectivity Mediates Motor Inhibitory Biomarker in Schizophrenia. Biol Psychiatry, 85:49-59. 30126607 10.1016/j.biopsych.2018.06.007 PMC6289820

[7] He K, Guo C, Guo M, Tong S, Zhang Q, Sun H, et al. (2019). Identification of serum microRNAs as diagnostic biomarkers for schizophrenia. Hereditas, 156:23. 31297041 10.1186/s41065-019-0099-3 PMC6598381

[8] Mazumder S, Datta S, Ray JG, Chaudhuri K, Chatterjee R (2019). Liquid biopsy: miRNA as a potential biomarker in oral cancer. Cancer Epidemiol, 58:137-145. 30579238 10.1016/j.canep.2018.12.008

[9] Agrawal S, Tapmeier TT, Rahmioglu N, Kirtley S, Zondervan KT, Becker CM (2018). The miRNA Mirage: How Close Are We to Finding a Non-Invasive Diagnostic Biomarker in Endometriosis? A Systematic Review. International Journal of Molecular Sciences, 19. 10.3390/ijms19020599 PMC5855821 29463003

[10] Grace AA (2016). Dysregulation of the dopamine system in the pathophysiology of schizophrenia and depression. Nat Rev Neurosci, 17:524-532. 27256556 10.1038/nrn.2016.57 PMC5166560

[11] Howes OD, McCutcheon R, Owen MJ, Murray RM (2017). The Role of Genes, Stress, and Dopamine in the Development of Schizophrenia. Biol Psychiatry, 81:9-20. 27720198 10.1016/j.biopsych.2016.07.014 PMC5675052

[12] George SR, Kern A, Smith RG, Franco R (2014). Dopamine receptor heteromeric complexes and their emerging functions. Dopamine, 211:183-200. 10.1016/B978-0-444-63425-2.00008-8 24968781

[13] Seeman P (2013). Schizophrenia and dopamine receptors. Eur Neuropsychopharmacol, 23:999-1009. 23860356 10.1016/j.euroneuro.2013.06.005

[14] Perreault ML, O'Dowd BF, George SR (2011). Dopamine receptor homooligomers and heterooligomers in schizophrenia. CNS Neurosci Ther, 17:52-57. 21199449 10.1111/j.1755-5949.2010.00228.x PMC3802522

[15] Razakarivony O, Newman-Tancredi A, Zimmer L (2021). Towards in vivo imaging of functionally active 5-HT1A receptors in schizophrenia: concepts and challenges. Transl Psychiatry, 11:22. 33414418 10.1038/s41398-020-01119-3 PMC7791062

[16] Vitucci D, Di Giorgio A, Napolitano F, Pelosi B, Blasi G, Errico F, et al. (2016). Rasd2 Modulates Prefronto-Striatal Phenotypes in Humans and 'Schizophrenia-Like Behaviors' in Mice. Neuropsychopharmacology, 41:916-927. 26228524 10.1038/npp.2015.228 PMC4707838

[17] Stilling RM, Benito E, Gertig M, Barth J, Capece V, Burkhardt S, et al. (2014). De-regulation of gene expression and alternative splicing affects distinct cellular pathways in the aging hippocampus. Front Cell Neurosci, 8:373. 25431548 10.3389/fncel.2014.00373 PMC4230043

[18] Quinlan AR (2014). BEDTools: The Swiss-Army Tool for Genome Feature Analysis. Curr Protoc Bioinformatics, 47: 11 12 11-34. 10.1002/0471250953.bi1112s47 PMC4213956 25199790

[19] Huang da W, Sherman BT, Lempicki RA (2009). Systematic and integrative analysis of large gene lists using DAVID bioinformatics resources. Nat Protoc, 4:44-57. 19131956 10.1038/nprot.2008.211

[20] Griffiths-Jones S, Saini HK, van Dongen S, Enright AJ (2008). miRBase: tools for microRNA genomics. Nucleic Acids Res, 36:D154-158. 17991681 10.1093/nar/gkm952 PMC2238936

[21] Shannon P, Markiel A, Ozier O, Baliga NS, Wang JT, Ramage D, et al. (2003). Cytoscape: a software environment for integrated models of biomolecular interaction networks. Genome Res, 13:2498-2504. 14597658 10.1101/gr.1239303 PMC403769

[22] Yu J, Qi D, Xing M, Li R, Jiang K, Peng Y, et al. (2011). MK-801 induces schizophrenic behaviors through downregulating Wnt signaling pathways in male mice. Brain Res, 1385:281-292. 21338586 10.1016/j.brainres.2011.02.039

[23] Shao ZC, Noh H, Kim WB, Ni P, Nguyen C, Cote SE, et al. (2019). Dysregulated protocadherin-pathway activity as an intrinsic defect in induced pluripotent stem cell-derived cortical interneurons from subjects with schizophrenia. Nature Neuroscience, 22:229-+. 30664768 10.1038/s41593-018-0313-z PMC6373728

[24] Chen Y, Tian L, Zhang F, Liu C, Lu T, Ruan Y, et al. (2013). Myosin Vb gene is associated with schizophrenia in Chinese Han population. Psychiatry research, 207:13-18. 23561489 10.1016/j.psychres.2013.02.026

[25] Vitucci D, Di Giorgio A, Napolitano F, Pelosi B, Blasi G, Errico F, et al. (2015). Rasd2 Modulates Psychotomimetic Drug Effects in Mice and Schizophrenia-related Phenotypes in Humans. European Psychiatry, 30.

[26] Bosia M, Bechi M, Pirovano A, Buonocore M, Lorenzi C, Cocchi F, et al. (2014). COMT and 5-HT1A-receptor genotypes potentially affect executive functions improvement after cognitive remediation in schizophrenia. Health Psychol Behav Med, 2:509-516. 25750798 10.1080/21642850.2014.905206 PMC4346068

[27] Mao P, Cui D, Zhao XD, Ma YY (2015). Prefrontal dysfunction and a monkey model of schizophrenia. Neurosci Bull, 31:235-241. 25822218 10.1007/s12264-014-1506-4 PMC5563701

[28] Chen HY, Fan Y, Zhao L, Hao Y, Zhou XJ, Guan YT, et al. (2017). Successful treatment with risperidone increases 5-HT 3A receptor gene expression in patients with paranoid schizophrenia - data from a prospective study. Brain and Behavior, 7. 10.1002/brb3.798 PMC5607560 28948091

[29] Akhondzadeh S (2001). The 5-HT hypothesis of schizophrenia. IDrugs, 4:295-300. 16025390

[30] Mitazaki S, Nakagawasai O, Onogi H, Watanabe K, Takahashi K, Tan-No K, et al. (2020). Role of prefrontal cortical 5-HT2A receptors and serotonin transporter in the behavioral deficits in post-pubertal rats following neonatal lesion of the ventral hippocampus. Behavioural Brain Research, 377. 10.1016/j.bbr.2019.112226 31521737

[31] Lecomte F, Aerts J, Plenevaux A, Defraiteur C, Chapuis-Hugon F, Rozet E, et al. (2020). Performance evaluation of a MIP for the MISPE-LC determination of p-[F-18]MPPF and a potential metabolite in human plasma. Journal of Pharmaceutical and Biomedical Analysis, 180. 10.1016/j.jpba.2019.113015 31865206

[32] Yang X, Zhou G, Ren T, Li H, Zhang Y, Yin D, et al. (2012). beta-Arrestin prevents cell apoptosis through pro-apoptotic ERK1/2 and p38 MAPKs and anti-apoptotic Akt pathways. Apoptosis, 17:1019-1026. 22699970 10.1007/s10495-012-0741-2

[33] Wu X, Xu FL, Xia X, Wang BJ, Yao J (2020). MicroRNA-15a, microRNA-15b and microRNA-16 inhibit the human dopamine D1 receptor expression in four cell lines by targeting 3'UTR -12 bp to + 154 bp. Artif Cells Nanomed Biotechnol, 48:276-287. 31858826 10.1080/21691401.2019.1703729

[34] Dai Y, Sa R, Guan F, Wang Q, Li Y, Zhao H (2021). A Purification Method of (18)F-FP-(+)-DTBZ via Solid-Phase Extraction With Combined Cartridges. Front Med (Lausanne), 8:693632. 34307421 10.3389/fmed.2021.693632 PMC8298858

[35] Kandeel M, Kitade Y (2013). Computational analysis of siRNA recognition by the Ago2 PAZ domain and identification of the determinants of RNA-induced gene silencing. PLoS One, 8:e57140. 23441235 10.1371/journal.pone.0057140 PMC3575500

[36] Carvelli L, Blakely RD, DeFelice LJ (2008). Dopamine transporter/syntaxin 1A interactions regulate transporter channel activity and dopaminergic synaptic transmission. Proc Natl Acad Sci U S A, 105:14192-14197. 18768815 10.1073/pnas.0802214105 PMC2528871

[37] Uchiumi O, Kasahara Y, Fukui A, Hall FS, Uhl GR, Sora I (2013). Serotonergic involvement in the amelioration of behavioral abnormalities in dopamine transporter knockout mice by nicotine. Neuropharmacology, 64:348-356. 22809709 10.1016/j.neuropharm.2012.07.016 PMC3586235

[38] Ma J, Shang S, Wang J, Zhang T, Nie F, Song X, et al. (2018). Identification of miR-22-3p, miR-92a-3p, and miR-137 in peripheral blood as biomarker for schizophrenia. Psychiatry Res, 265:70-76. 29684772 10.1016/j.psychres.2018.03.080

[39] Backes C, Meese E, Keller A (2016). Specific miRNA Disease Biomarkers in Blood, Serum and Plasma: Challenges and Prospects. Mol Diagn Ther, 20:509-518. 27378479 10.1007/s40291-016-0221-4

[40] Rodriguiz RM, Nadkarni V, Means CR, Pogorelov VM, Chiu YT, Roth BL, et al. (2021). LSD-stimulated behaviors in mice require beta-arrestin 2 but not beta-arrestin 1. Sci Rep, 11:17690. 34480046 10.1038/s41598-021-96736-3 PMC8417039

[41] Ghiglieri V, Napolitano F, Pelosi B, Schepisi C, Migliarini S, Di Maio A, et al. (2015). Rhes influences striatal cAMP/PKA-dependent signaling and synaptic plasticity in a gender-sensitive fashion. Sci Rep, 5:10933. 26190541 10.1038/srep10933 PMC4507147

[42] Beaulieu JM, Sotnikova TD, Marion S, Lefkowitz RJ, Gainetdinov RR, Caron MG (2005). An Akt/beta-arrestin 2/PP2A signaling complex mediates dopaminergic neurotransmission and behavior. Cell, 122:261-273. 16051150 10.1016/j.cell.2005.05.012

[43] Cheng CJ, Bahal R, Babar IA, Pincus Z, Barrera F, Liu C, et al. (2015). MicroRNA silencing for cancer therapy targeted to the tumour microenvironment. Nature, 518:107-110. 25409146 10.1038/nature13905 PMC4367962

[44] Hu YB, Zhang YF, Wang H, Ren RJ, Cui HL, Huang WY, et al. (2019). miR-425 deficiency promotes necroptosis and dopaminergic neurodegeneration in Parkinson's disease. Cell Death Dis, 10:589. 31383850 10.1038/s41419-019-1809-5 PMC6683159

[45] Wang Y, Wang L (2017). miR-34a attenuates glioma cells progression and chemoresistance via targeting PD-L1. Biotechnol Lett, 39:1485-1492. 28721584 10.1007/s10529-017-2397-z

[46] Lu J, Getz G, Miska EA, Alvarez-Saavedra E, Lamb J, Peck D, et al. (2005). MicroRNA expression profiles classify human cancers. Nature, 435:834-838. 15944708 10.1038/nature03702

