# Supplementary material for: MiR-4763-3p targeting RASD2as a Potential Biomarker and Therapeutic Target for Schizophrenia
Source: Aging Dis. 2022 Jul 11;13(4):1278–92. doi: 10.14336/AD.2022.0103 (PMC9286908; doi:10.14336/AD.2022.0103)
Supplement: Supplementary file 1 [file AD-13-4-1278-s.pdf]

SUPPLEMENTARY DATA

MiR-4763-3p targeting *RASD2* as a Potential Biomarker and Therapeutic Target for Schizophrenia

Jiao Wang[1*], Wenxin Qi[1], Hongwei Shi[1], Lin Huang[1], Fujiang Ning[8], Fushuai Wang[1], Kai Wang[7], Haotian Bai[2], Hao Wu[1], Junyi Zhuang[1], Huanle Hong[1], Haicong Zhou[1], Hu Feng[1], Yinping Zhou[1], Naijun Dong[1], Li Liu[8], Yanyan Kong[3*], Jiang Xie[2*], Robert Chunhua Zhao[1,4,5,6*]

© 2021. Wang J. et al. Published online at http://www.aginganddisease.org/EN/10.14336/AD.2022.0103

SUPPLEMENTARY DATA

A

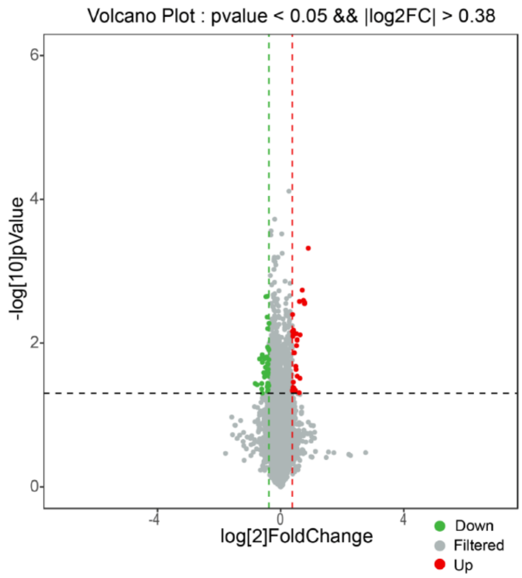

B

miRNA	Common target	MFE (kcal/mol)	p-value
hsa-miR-328-3p	CLEC18B	-41.1	0.000165
hsa-miR-5006-5p	CLEC18B	-33.9	0.003139
hsa-miR-6804-3p	CLEC18B	-34.3	0.004086
hsa-miR-7113-3p	CLEC18B	-42.1	0.000180
hsa-miR-328-3p	CLEC18C	-41.1	0.000165
hsa-miR-5006-5p	CLEC18C	-33.9	0.003139
hsa-miR-6804-3p	CLEC18C	-34.3	0.004086
hsa-miR-7113-3p	CLEC18C	-42.1	0.000180
hsa-miR-1266-5p	NAPRT	-34.1	0.004915
hsa-miR-211-3p	NAPRT	-32.9	0.004784
hsa-miR-3944-3p	NAPRT	-39.1	0.000728
hsa-miR-6757-5p	NAPRT	-32.8	0.007730
hsa-miR-1185-2-3p	NCR3LG1	-31.6	0.008461
hsa-miR-4530	NCR3LG1	-33.3	0.002399
hsa-miR-4701-5p	NCR3LG1	-33.5	0.004962
hsa-miR-619-5p	NCR3LG1	-38.1	0.000344
hsa-miR-483-3p	SPDYE2	-39.6	0.000167
hsa-miR-6810-3p	SPDYE2	-37.2	0.001418
hsa-miR-6845-3p	SPDYE2	-39.0	0.000364
hsa-miR-760	SPDYE2	-31.9	0.008904
hsa-miR-483-3p	SPDYE2B	-39.6	0.000167
hsa-miR-6810-3p	SPDYE2B	-37.2	0.001418
hsa-miR-6845-3p	SPDYE2B	-39.0	0.000364
hsa-miR-760	SPDYE2B	-31.9	0.008904

Supplementary Figure 1. *RASD2* as the hub gene among the DEGs between patients with schizophrenia and healthy controls. (A) Volcano plot showing 21 upregulated genes and 40 downregulated genes in schizophrenia. **(B)** miRNA-mRNA interactions predicted by RNAhybrid and BLASTN, showing values for protein-coding genes regulated by at least one of four (or possibly more) different miRNAs. MFE: minimum free energy. Data represent the mean ± SEM, n ≥ 3; *p < 0.05, **p < 0.01, ***p < 0.001.

© 2021. Wang J. et al. Published online at http://www.aginganddisease.org/EN/10.14336/AD.2022.0103

SUPPLEMENTARY DATA

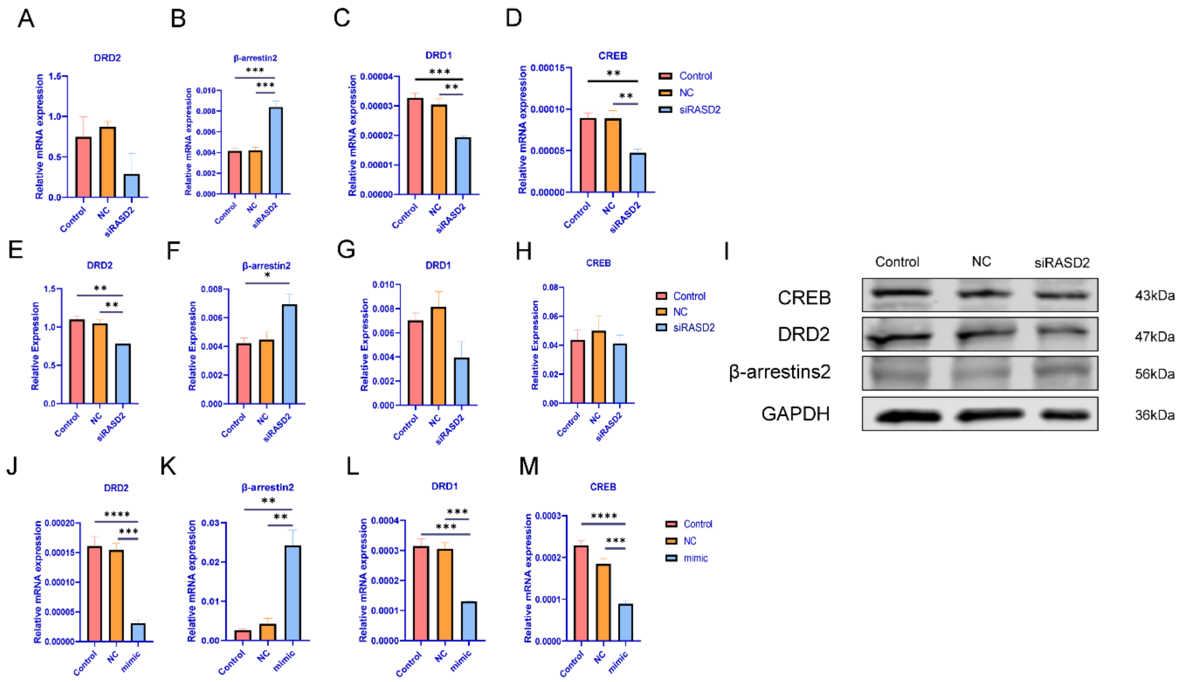

Supplementary Figure 2. miRNA-4763-3p is involved in the D1- and D2-dependent signaling pathways by regulating the target gene *RASD2*. (A–D) Expression of mRNAs encoding *DRD2, β-arrestin2, DRD1*, and *CREB* in N2A cells transfected with si*RASD2*, the corresponding negative control (NC), or control as assessed with qPCR, and expression of the proteins **(E–I)** as assessed with western blotting. **(J–M)** Expression of mRNAs encoding *DRD2, β-arrestin2, DRD1*, and *CREB* in N2A cells transfected with a miR-4763-3p mimic, the corresponding NC, or control as assessed with qPCR. Data represent the mean ± SEM, $n \geq 3$; *$p < 0.05$, **$p < 0.01$, ***$p < 0.001$, ****$p < 0.0001$.

© 2021. Wang J. et al. Published online at http://www.aginganddisease.org/EN/10.14336/AD.2022.0103
